# An Isolated Cutaneous Nodule: Dupilumab-Induced Sarcoid-Like Reaction

**DOI:** 10.7759/cureus.68061

**Published:** 2024-08-28

**Authors:** Gina N Bash, Jina Chung, Nicole Fett

**Affiliations:** 1 Department of Dermatology, Oregon Health & Science University, Portland, USA; 2 Department of Dermatology, University of Pennsylvania, Philadelphia, USA

**Keywords:** sarcoid, nodule, dupilumab, granuloma, drug-induced sarcoid-like reaction

## Abstract

Dupilumab has recently been recognized as a potential trigger for drug-induced sarcoid-like reactions (DISR). This phenomenon may become more prevalent with increased utilization of this drug for a multitude of skin and atopic conditions. We present a unique case of a patient developing a solitary cutaneous nodule on her left forearm following dupilumab initiation. Histopathology and MRI studies confirmed that this nodule had features of a sarcoid granuloma. Six months following dupilumab discontinuation, the patient's granuloma resolved. This case demonstrates that dupilumab can induce cutaneous-limited autoimmune disease and stresses the importance of prompt recognition of dupilumab-induced sarcoid-like reactions for appropriate diagnosis and treatment.

## Introduction

Dupilumab has been recently implicated in drug-induced sarcoid-like reactions (DISR) in a limited number of patients started on the medication [1-3]. Dupilumab is a monoclonal antibody interleukin-4 receptor (IL-4R) alpha antagonist that inhibits IL-4 and IL-13 signaling. It is currently FDA-approved to treat dermatologic conditions including atopic dermatitis, asthma, and prurigo nodularis. Although dupilumab generally has a favorable side effect profile, the emergence of new cases of sarcoid-like reactions warrants further characterization. Here, we report the case of a 57-year-old woman who developed a singular new subcutaneous granulomatous tumor on her left forearm following initiation of dupilumab treatment for prurigo nodularis and atopic dermatitis.

## Case presentation

A 57-year-old female patient with a history of atopic dermatitis, asthma, and prurigo nodules presented to a dermatology clinic in April of 2023 with a four-month history of an asymptomatic, large, subcutaneous tumor on the left forearm. Seven months prior, she was started on 300 mg dupilumab every 14 days with clearance of her prurigo nodules and a decrease of her atopic dermatitis from 15% body surface area (BSA) to 1%. 

**Figure 1:**
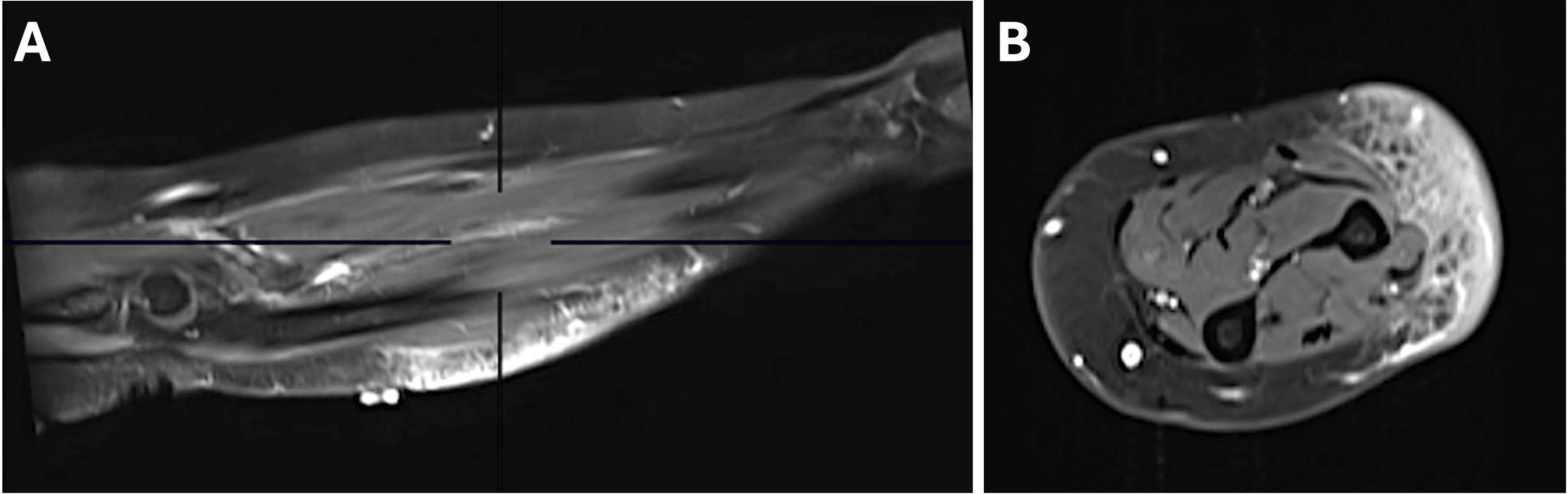
Enhancement on MRI views of the left forearm. Coronal (A) and axial (B) MRI views of the left forearm demonstrate ill-defined diffuse skin and subcutaneous adipose tissue thickening, T2 hyperintense edema, and diffuse enhancement of the ulnar forearm.

**Figure 2:**
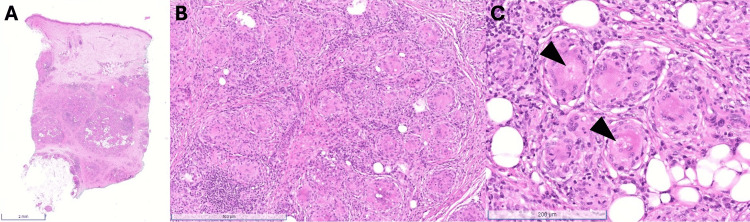
Histopathology showing nodular granulomatous dermatitis with asteroid bodies. (A) Punch biopsy (H&E) of the dermis and subcutis. (B) A higher-power view demonstrates nodular aggregates of histiocytes forming granulomas. (C) Multinucleated giant cells and asteroid bodies (arrowheads) are present in the biopsy.

On examination, the patient had a 10×5 cm deep, very firm tumor on the left forearm without overlying skin changes. MRI with contrast revealed diffuse, confluence plaque-like skin and subcutaneous tissue thickening, edema, and enhancement along the ulnar forearm (Figure 1A-1B). A 6 mm punch biopsy revealed multiple nodular aggregates of histiocytes forming naked granulomas in the dermis and subcutis with multinucleated giant cells and asteroid bodies (Figure 2). Periodic acid-Schiff (PAS), acid-fast bacteria (AFB), and Fite's acid-fast stains were negative for organisms, and no refractile foreign bodies were observed. These histopathologic findings were consistent with sarcoidosis. Her review of systems was otherwise negative, and she had a normal chest X-ray.

Dupilumab was discontinued at that time. The nodule was 3×1 cm at the two-month follow-up and completely resolved by the six-month follow-up. Given the time course in relationship to the initiation of dupilumab and resolution with dupilumab cessation as the only treatment, this patient's lesion was most likely a cutaneous form of DISR.

Thus far, patient follow-up revealed no further nodules since stopping dupilumab treatment. Unfortunately, however, her atopic dermatitis worsened after dupilumab discontinuation. She was started on a total of 15 mg methotrexate weekly with some improvement in her itch and prurigo nodules but persistent uncomfortable abdominal pain and diarrhea. She was subsequently switched to subcutaneous methotrexate 20 mg once a week. Despite this modification, her atopic dermatitis continued to flare, and she had gastrointestinal side effects to the methotrexate resulting in the cessation of therapy. She was most recently started on ruxolitinib 1.5% cream twice daily, limiting to 20% BSA or less, and narrowband UVB phototherapy twice weekly with some improvement in her atopic dermatitis. She is currently being followed closely in the clinic. 

## Discussion

This is the first reported presentation of an isolated cutaneous tumor consistent with DISR from dupilumab. Published cases have reported patients on dupilumab developing systemic sarcoid-like granulomatosis affecting the central nervous system including meningoencephalitis [2] and neuro-sarcoidosis-like reaction with temporoparietal lobe granulomas and brain edema [3]. In the first case, a 28-year-old male presented four months after dupilumab initiation for severe AD with confusion, headaches, emesis, and photophobia. His imaging revealed micronodular brain and lung infiltration. The timing of granuloma development after dupilumab initiation was similar in both our case and the one described. However, unlike in our case, this patient had a much more severe and systemic presentation rather than a single granuloma. In the second case, the patient was a 79-year-old man who also presented with neurological symptoms including visual hallucinations, disorientation, and behavioral changes. He had been on dupilumab for four months. MRI showed T1 contrast-enhancing lesions present in the right temporoparietal lobe. In addition, his cerebrospinal fluid (CSF) analysis demonstrated lymphocytic pleocytosis and elevated protein. In this case, the presentation was localized to the brain and CSF, with corresponding symptoms. As noted in this report, DISR cannot be clinically differentiated from sarcoidosis, but rather correlation of symptoms with dupilumab treatment initiation and resolution after drug withdrawal are indicative of DISR. The differences in severity of disease presentation in these cases are likely due to patient-specific immune systems and subsequent responses, but given the paucity of reports, there is currently limited supporting data [4]. 

Another recent case described non-caseating granulomas in the lungs and skin of a 34-year-old woman after seven months of dupilumab treatment for eosinophilic rhinosinusitis [1]. The patient noticed a small 10 mm subcutaneous nodule in her right upper extremity seven months after dupilumab initiation. Similar to our patient, this patient did not present with systemic symptoms, although she was found to have additional bilateral pulmonary nodules and multiple mediastinal lymphadenopathies along with ocular granulomas. Again her presentation was consistent with a sarcoidosis picture; however, with the timing of dupilumab and disease onset, dupilumab was deemed the causative agent. She was switched to mepolizumab, an IL-5 inhibitor, and her DISR resolved.

Unlike those cases of more widespread reactions, which reported using corticosteroids [2,3] or switching to targeted therapy, mepolizumab [1], the granulomatous tumor in our patient resolved with discontinuation of dupilumab alone. In line with previous reports, resolution upon dupilumab withdrawal in combination with the presence of a nodular granulomatous dermatitis on histopathology strongly points to a DISR as a result of dupilumab treatment in this case rather than the de novo development of sarcoidosis. 

Although the mechanism of DISR has not been fully elucidated, some evidence supports that disturbance of the Th1/Th2 balance by the dupilumab pathway may encourage granuloma formation [4]. By blocking IL-4 and IL-13, dupilumab effectively decreases Th2 T-cell differentiation and therefore favors the Th1 pathway. Th1-derived cytokines such as IL-2 and INF-γ and their effects on macrophage polarization may play a role in immune response and granuloma development [5]. Although macrophage polarization simplifies the rather complex cell-cell interactions involved in immune regulation and may limit a more nuanced understanding of immune cytokine crosstalk, it does suggest an explanation for the development of sarcoid-like granulomas following the inhibition of the Th2 pathway. Other drugs implicated in causing DISR include antiretroviral therapy, TNF-α antagonists, interferon therapy, and immune checkpoint inhibitors [6]. 

## Conclusions

Dupilumab is capable of inducing isolated cutaneous tumors through a DISR. With recent reports of DISR after starting dupilumab and an increase in the use of this drug for multiple atopic diseases, this entity will likely become more prevalent. Prompt recognition is crucial to ensure appropriate diagnosis and treatment. Discontinuing dupilumab appears to reverse the sarcoid-like reaction in mild cases and may be the most appropriate next step in patients with cutaneous-limited disease, though further studies would strengthen this observation.
